# Tai Chi Chuan and Baduanjin Mind-Body Training Changes Resting-State Low-Frequency Fluctuations in the Frontal Lobe of Older Adults: A Resting-State fMRI Study

**DOI:** 10.3389/fnhum.2017.00514

**Published:** 2017-10-30

**Authors:** Jing Tao, Xiangli Chen, Jiao Liu, Natalia Egorova, Xiehua Xue, Weilin Liu, Guohua Zheng, Ming Li, Jinsong Wu, Kun Hu, Zengjian Wang, Lidian Chen, Jian Kong

**Affiliations:** ^1^College of Rehabilitation Medicine, Fujian University of Traditional Chinese Medicine, Fuzhou, China; ^2^Fujian Key Laboratory of Rehabilitation Technology, Fujian University of Traditional Chinese Medicine, Fuzhou, China; ^3^Department of Psychiatry, Massachusetts General Hospital and Harvard Medical School, Charlestown, MA, United States; ^4^Department of Rehabilitation Psychology and Special Education, University of Wisconsin-Madison, Madison, WI, United States; ^5^Affiliated Rehabilitation Hospital, Fujian University of Traditional Chinese Medicine, Fuzhou, China; ^6^Developmental and Educational Psychology, South China Normal University, Guangzhou, China

**Keywords:** mind-body exercise, memory, aging, fractional amplitude of low-frequency fluctuations (fALFF), resting-state functional magnetic resonance imaging (fMRI), frequency bands

## Abstract

Age-related cognitive decline is a significant public health concern. Recently, non-pharmacological methods, such as physical activity and mental training practices, have emerged as promising low-cost methods to slow the progression of age-related memory decline. In this study, we investigated if Tai Chi Chuan (TCC) and Baduanjin modulated the fractional amplitude of low-frequency fluctuations (fALFF) in different frequency bands (low-frequency: 0.01–0.08 Hz; slow-5: 0.01–0.027 Hz; slow-4: 0.027–0.073 Hz) and improved memory function. Older adults were recruited for the randomized study. Participants in the TCC and Baduanjin groups received 12 weeks of training (1 h/day for 5 days/week). Participants in the control group received basic health education. Each subject participated in memory tests and fMRI scans at the beginning and end of the experiment. We found that compared to the control group: (1) TCC and Baduanjin groups demonstrated significant improvements in memory function; (2) TCC increased fALFF in the dorsolateral prefrontal cortex (DLPFC) in the slow-5 and low-frequency bands; and (3) Baduanjin increased fALFF in the medial PFC in the slow-5 and low-frequency bands. This increase was positively associated with memory function improvement in the slow-5 and low-frequency bands across the TCC and Baduanjin groups. Our results suggest that TCC and Baduanjin may work through different brain mechanisms to prevent memory decline due to aging.

## Introduction

Age is the main risk factor for most common neurodegenerative diseases, such as mild cognitive impairment (MCI) and Alzheimer’s disease (AD). Memory dysfunction is the primary cognitive symptom in MCI and AD and has a profound impact on those whom it affects (McKhann et al., 2011). Nevertheless, pharmaceutical treatments for age-related memory decline remain unsatisfactory.

Recently, non-pharmacological methods, such as physical activity and mental training practices, have emerged as promising low-cost methods to slow the progression of age-related memory decline (Hillman et al., 2008; Erickson et al., 2011, 2014; Killgore et al., 2013; Makizako et al., 2013; Voss et al., 2013; Kelly et al., 2014; Tang and Posner, 2014; Tamura et al., 2015). For instance, Ruscheweyh et al. (2011) found that a 6 months intervention of low-intensity physical activity can improve episodic memory performance in healthy elderly individuals, and this improvement is associated with increases in local gray matter volume in the prefrontal and cingulate cortex, and Brain-derived neurotrophic factor (BDNF) levels. Innes et al. (2017) also reported that 12 min/day for 3 months of Kirtan Kriya meditation training can significantly improve memory and cognitive performance. Unlike pharmaceutical treatments, these methods usually lack serious side effects.

Tai Chi Chuan (TCC) and Baduanjin are popular mind-body practices (Wang et al., 2010; Zheng et al., 2014; Tao et al., 2015). Both of these practices combine meditation with slow movements, deep breathing, and relaxation to smooth vital energy (or qi) flow in the body (Wang et al., 2010). However, these practices are also different from each other; TCC involves more complicated body movements and requires moving one’s trunk and all four limbs (Wei et al., 2013), whereas the movement involved in Baduanjin is much simpler and is characterized by eight fixed movements (Xiong et al., 2015). Accumulating evidence has shown that TCC and Baduanjin practice improves cognitive performance and memory function (Wang, 2007; Chang et al., 2010; Lam et al., 2011; Tsai et al., 2013; Fong et al., 2014; Li F. et al., 2014; Wayne et al., 2014; Yin et al., 2014; Zheng et al., 2015). Nevertheless, the mechanisms underlying TCC and Baduanjin are still poorly understood.

In recent years, spontaneous fluctuations in brain activity during rest have drawn the attention of neuroimaging researchers. Investigators believe that these slow-frequency fluctuations may provide information about the intrinsic functional organization of the brain (Fox and Raichle, 2007). Furthermore, studies suggest that the human brain is a complex system that can generate a multitude of oscillatory waves, with different oscillatory classes carrying different dimensions of brain integration. The coupling of different bands of oscillators can provide enhanced combinatorial opportunities for storing complex temporal patterns to accomplish specific functions (Knyazev, 2007).

The low frequency fluctuations (LFF) between 0.01 Hz and 0.08 Hz are of particular relevance to resting state fMRI (rs-fMRI; Biswal et al., 1995). This low frequency range has been further divided into several distinct bands (Buzsáki and Draguhn, 2004), such as slow-4 (0.027–0.073 Hz) and slow-5 (0.01–0.027 Hz), which may indicate the modulation of cortical excitability and neuronal synchronization (Hoptman et al., 2010; Zuo et al., 2010). Recently, investigators have analyzed resting-state fMRI data filtered at the slow-4 and slow-5 bands separately to investigate AD (Liu et al., 2014), MCI (Han et al., 2012; Zhao et al., 2015), social anxiety disorder (SAD; Zhang et al., 2015), Parkinson’s Disease (PD; Esposito et al., 2013), and schizophrenia (Hoptman et al., 2010). Studies have found characteristic differences between these specific bands, further endorsing the value of distinguishing the slow-4 and slow-5 bands.

There are many methods that can be used to investigate the brain’s resting state spontaneous fluctuations. One such method is to characterize the regional spontaneous neuronal activity using the fractional amplitude of low frequency fluctuations (fALFF; Zang et al., 2007; Zou et al., 2008). As a normalized index of amplitude of low frequency fluctuations (ALFF), fALFF is defined as the total power within the low-frequency range divided by the total power in the entire detectable frequency range (Zuo et al., 2010). This method significantly suppresses non-specific signal components in resting state MRI and increases sensitivity to regional spontaneous brain activity (Zuo et al., 2010).

Alterations in fALFF have been found in several diseases. For instance, Sui et al. (2015) reported that in schizophrenic patients, increased cognitive performance was associated with higher fALFF in the striatum and decreased cognitive performance was associated with higher fALFF in the dorsolateral prefrontal cortex (DLPFC). McGill et al. (2014) reported decreased fALFF in the (PFC) and thalamus in patients with idiopathic generalized epilepsy. Additionally, Han et al. (2011) found that MCI is associated with decreased ALFF/fALFF values in the PCC/PCu, mPFC, hippocampus/PHG and prefrontal regions and increased ALFF/fALFF values in the occipital and temporal regions.

In this study, we investigated changes in spontaneous brain activity using fALFF in older adults following 3-months of TCC or Baduanjin practice. We hypothesized that 3-months of TCC and Baduanjin practice would improve memory function and modulate spontaneous brain activity in the brain regions associated with memory. In addition, we hypothesized that the modulatory effects of TCC and Baduanjin might vary in different low frequency bands.

## Materials and Methods

In this study, we applied a data driven method to investigate fALFF changes before and after TCC and Baduanjin as compared to a control group. Although, the data has been used previously to investigate the resting state functional connectivity changes of the hippocampus (Tao et al., 2016) and DLPFC (Tao et al., 2017a) and brain structure changes (Tao et al., 2017b) following TCC and Baduanjin practice, we have never reported the results published in this manuscript. Please also see these published studies for more details on the experimental procedure.

### Participants

The study was approved by the Medical Ethics Committee of Affiliated Rehabilitation Hospital, Fujian University of Traditional Chinese Medicine and registered in the Chinese Clinical Trial Registry (ChiCTR^1^, ChiCTR-IPR-15006131). All participants were informed and signed a written consent.

Two cohorts of older adults from one community were recruited independently and randomized into a TCC or control group in one cohort and a Baduanjin or control group in the other cohort. We recruited the two cohorts separately to avoid potential cross practicing between TCC and Baduanjin. The randomized treatment assignments were sealed in opaque envelopes and opened each time when new participants were included. Outcome raters were blind to the group allocation.

Inclusion criteria were: (1) 50–70 years old; (2) right-handed; and (3) no regular physical exercise for at least 1 year (the minimal standard for regular physical exercise was defined as 30 min 3–4 times per week for the past 3 months). Exclusion criteria were: (1) history of stroke; (2) suffered from severe cerebrovascular disease, musculoskeletal system disease, or other contraindications caused by sports injury; (3) a score of Beck depression inventory (BDI-II) ≥ 14 (Beck et al., 1996); and (4) a score on the Mini-Mental State Exam (MMSE) < 24 (Zhang et al., 1990).

### Intervention

Two professional instructors with more than 5 years of training experience from Fujian University of Traditional Chinese Medicine were responsible for TCC and Baduanjin exercise training. To guarantee research quality, two staff members monitored the whole training procedure.

#### Tai Chi Chuan Exercise Group

TCC exercise, which was based on Yang-style 24-form (China National Sports Commission, 1983), was conducted for 60 min per session, 5 days per week for 12 weeks. Each session consisted of a warm-up and review of Tai Chi principles, TCC exercises, breathing technique training, and relaxation.

#### Baduanjin Exercise Group

The Baduanjin training regimen was in accordance with “Health Qigong—Baduanjin”, published by the General Administration of Sport of China. Each Baduanjin session consisted of a warm-up, eight fixed movements, and ending posture. The frequency of the Baduanjin exercise was the same as the TCC group, i.e. 60 min per session, one session per day, 5 days per week for 12 weeks.

#### Control Group

Participants in the control group received basic health education at the beginning of the experiment (Hughes et al., 2014). For the next 12 weeks, they were instructed to keep their original physical activity habits. At the end of the experiment (after the second MRI scan), free TCC or Baduanjin training was offered.

### Behavioral Measurement

The Wechsler Memory Scale–Chinese Revision (WMS-CR) was used to assess the memory function of each participant. The WMS-CR is designed to assess memory function (Gong and Wang, 1989; Woodard and Axelrod, 1995) and is one of the most frequently used clinical assessments. It consists of ten subtests: information, orientation, mental control, picture, recognition, visual reproduction, associative learning, touch, comprehension memory, and digit span. It also provides an overall memory quotient (MQ). Two licensed WMS-CR raters who were blinded to the randomization distribution administered the WMS-CR.

### MRI Acquisition

All MRI scans were acquired on a 3.0T magnetic resonance scanner (General Electric SignaHDxt, Milwaukee, WI, USA) with an 8-channel phased-array head coil. For the rs-fMRI, the scans were acquired with TR = 2100 ms, TE = 30 ms, flip angle = 90°, slice thickness = 3 mm, gap = 0.6 mm, acquisition matrix = 64 × 64, voxel size = 3.125 × 3.125 × 3.6 mm^3^, 42 axial slices, FOV = 200 × 200 mm, phases per location = 160. The scan lasted for 5 min and 36 s, and participants were required to stay awake with their eyes closed and ears plugged during the rs-fMRI scanning. In addition, magnetization-prepared rapid gradient echo (MPRAGE) T1-weighted images were collected.

### Statistical Analysis

#### Behavioral Analysis

Baseline characteristics were compared by one-way analysis of variance (ANOVA) and Chi square tests using SPSS 18.0 Software (SPSS Inc., Chicago, IL, USA). During the analysis, all control participants from the two cohorts were combined into one group to increase the power. In order to estimate the effects of TCC and Baduanjin, ANCOVA analysis was applied to compare the change of MQ and the subtests across the three groups with age (years), with gender and education (years) included as covariates in the model. *Post hoc* analysis (Sidak corrected) was applied to explore the between-group differences.

#### Resting State Data Analysis

The fMRI data preprocessing was performed using Data Processing Assistant for Resting-State fMRI (DPARSF) Software (available at: http://rfmri.org/DPARSF; Chao-Gan and Yu-Feng, 2010) in MATLAB (Mathworks Inc., Natick, MA, USA). The software is based on Statistical Parametric Mapping (SPM8)^2^ and the Resting-State fMRI Data Analysis Toolkit^3^ (Song et al., 2011).

The first 10 volumes of functional data for each subject were discarded for signal equilibrium and participants’ adaptation to the imaging noise. The remaining volumes were slice timing corrected, within-subject spatially realigned, co-registered to the respective structural images for each subject, and then segmented. Subjects were excluded if head movement exceeded 3 mm on any axis or if head rotation was greater than 3°. To perform subject-level correction of head motion, the Friston 24-parameter model (6 head motion parameters, 6 head motion parameters one time point before, and the 12 corresponding squared items; Friston et al., 1996; Yan et al., 2013) was used. Images were normalized using structural image unified segmentation and then re-sampled to 3-mm cubic voxels. After smoothing with a 6 mm full-width at half maximum (FWHM) Gaussian kernel, the linear and quadric trends of the time courses were removed. Similar to previous studies (Han et al., 2011), no temporal filtering was implemented during preprocessing so that the entire frequency band could be calculated. In this study, we applied three frequency bands: slow-5 (0.01–0.027 Hz), slow-4 (0.027–0.073 Hz), and the traditionally used low-frequency (0.01–0.08 Hz) bands.

Group analysis was performed with a random effects model using SPM8. To explore the difference between TCC and Baduanjin after longitudinal treatment, we used a full factorial module in SPM8 with two factors for group analysis. The first factor had three levels (TCC, Baduanjin, control group) and the second factor had two levels (pre- and post-treatment). Age, gender and years of education were also included in the analysis as covariates of non-interest. A threshold of a voxel-wise *p* < 0.001 uncorrected and cluster-level *p* < 0.05 family-wise error corrected based on the random Gaussian field theory base (Lindquist et al., 2009) was applied.

## Results

102 older adults between 50–70 years old were screened for this study. Of the 90 participants who were qualified for the study and finished baseline scans, 62 participants completed all study procedures (21 in the TCC group, 16 in the Baduanjin group, and 25 in the control group). Four participants in the TCC group dropped out (1 due to relocation, 1 due to unwillingness to get the second MRI scan, and 2 due to scheduling conflicts). Nine participants in the Baduanjin group dropped out (8 due to scheduling conflicts and 1 due to unwillingness to participate in the MRI scan). Fifteen participants in the control group dropped out (11 due to scheduling conflicts and 4 due to inability to participate in post-treatment MRI scans). One subject in the Baduanjin group was excluded from fALFF analysis due to excessive head movement (exceeded 3.0 mm).

### Behavioral Results

Group characteristics are shown in Table 1. Age, gender, handedness, average years of education, MMSE score, and BDI score did not significantly differ among the three groups (*P* > 0.05). Average attendance rates were 95% in the TCC group (ranging from 88% to 100%) and 97% in the Baduanjin group (ranging from 92% to 100%).

**Table 1:** Demographics of study participants and clinical outcome measurements.

				Between-group difference		
Characteristics	Control (*n* = 25) Mean (SD)	Tai Chi Chuan (*n* = 21) Mean (SD)	Baduanjin (*n* = 15) Mean (SD)	Tai Chi Chuan vs. Control *P* value	Baduanjin vs. Control *p* value	Tai Chi Chuan vs. Baduanjin *p* value
Age^†^	59.76 (4.83)	62.38 (4.55)	62.33 (3.88)	0.055	0.087	0.975
Gender (female/male)^‡^	19/6	13/8	9/6		0.473	
Handedness (right/left)	25/0	21/0	15/0	-	-	-
Average years of education^†^	8.52 (3.65)	9.61 (3.02)	9.13 (2.69)	0.255	0.563	0.658
MQ_Pre treatment^†^	99.08 (14.59)	105.81 (10.24)	99.20 (9.30)	0.065	0.976	0.112
MQ_Post treatment^†††^	97.76 (13.92)	123.57 (11.42)	124.86 (11.21)	<0.001	<0.001	0.907

*^†^p values were calculated with one-way analysis of variance, ^‡^p values were calculated with the chi-square test, ^†††^p values were calculated with mixed-model regression*.

MQ scores before and after exercise are presented in Table 1. No significant differences were found among the three groups at baseline. ANCOVA analysis of change between the baseline and post-treatment MQ scores showed a significant difference among the three groups (*F* = 25.45, *p* < 0.001). *Post hoc* Sidak correction analysis showed that compared with the control group, MQ scores significantly increased in the TCC and Baduanjin groups (Baduanjin: *p* < 0.001, TCC: *p* < 0.001). There were no significant differences between the TCC and Baduanjin groups (*p* = 0.233). The comparisons of the subscores of WMS-CR showed TCC significantly increased visual reproduction subscores compared to controls. Baduanjin produced greater improvement in mental control, recognition, visual reproduction, touch and comprehension memory subscores compared to controls after bonferroni correction (*p* < 0.0063). Baduanjin also produced greater improvements in touch subscores compared to TCC after bonferroni correction (*p* < 0.0063). Please also see our previous publications on subscore changes across different treatment (Tao et al., 2017b).

### Resting-State fMRI Data Analysis Results

#### fALFF in Low-Frequency Band (0.01–0.08 Hz)

**Figure 1:**
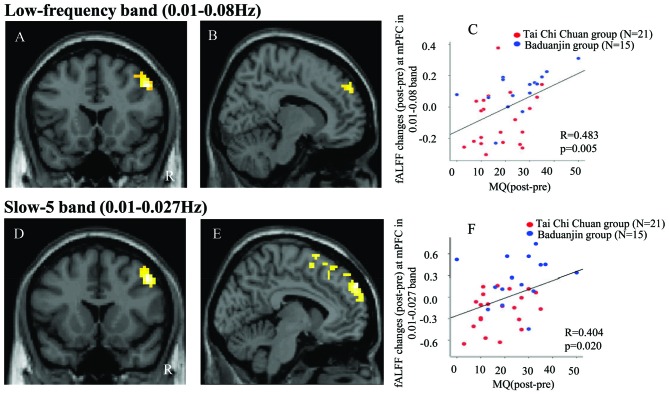
**(A)** Increased fractional amplitude of low-frequency fluctuations (fALFF) in the 0.01–0.08 Hz band in the Tai Chi Chuan (TCC) group compared with the control group. **(B)** Increased fALFF in the 0.01–0.08 Hz band in the Baduanjin group compared with the control group. **(D)** Increased fALFF in the 0.01–0.027 Hz band in the TCC group compared with the control group. **(E)** Increased fALFF in the 0.01–0.027 Hz band in the Baduanjin group compared with the control group. **(C,F)** Scatter plots showing the association between the prefrontal cortex fALFF value of the significant cluster and improvements in memory across the TCC and Baduanjin groups, corrected for age, gender, years of education (**C**: *r* = 0.483, *p* = 0.005; **F**: *r* = 0.404, *p* = 0.02). R: right.

Pre- and post-treatment comparison of fALFF in the low-frequency band (0.01–0.08 Hz) among the three groups showed that after 12 weeks, fALFF was significantly increased in the right DLPFC in the TCC group compared to the control group (Table 2, Figure 1A). In the Baduanjin group, there was a significant increase in fALFF in the bilateral medial prefrontal cortex (mPFC) compared to the control group (Table 2, Figure 1B). No significant difference was observed between the TCC and Baduanjin groups at the threshold we set.

**Table 2:** Comparisons of fractional amplitude of low-frequency fluctuations (fALFF) at different bands between groups.

		MNI coordinates					
Contrast	Brain regions	Cluster size	Peak *z*-value	Cluster effect size	*X*	*Y*	*Z*
**Low-frequency band**							
TaiChiChuan > control	R DLPFC	60	5.45	3.02	51	18	39
Baduanjin > control	L mPFC	33	4.64	1.68	−12	12	66
	R mPFC	34	4.23	1.59	12	54	36
Control > Tai Chi Chuan		No brain region above the threshold					
Control > Baduanjin		No brain region above the threshold					
Tai Chi Chuan > Baduanjin		No brain region above the threshold					
Baduanjin > Tai Chi Chuan		No brain region above the threshold					
**Slow-5 band**							
Tai Chi Chuan > control	R DLPFC	65	5.1	2.17	48	15	42
Baduanjin > control	R mPFC	167	4.51	1.67	9	57	36
	L mPFC		4.41		−6	60	39
Control > Tai Chi Chuan		No brain region above the threshold					
Control > Baduanjin		No brain region above the threshold					
Tai Chi Chuan > Baduanjin		No brain region above the threshold					
Baduanjin > Tai Chi Chuan		No brain region above the threshold					

*L, left; R, right; DLPFC, dorsolateral prefrontal cortex; mPFC, medial prefrontal cortex*.

To explore the difference between Tai Chi vs. Baduanjin intervention, we also applied a relatively less conservative threshold of voxel-wise *p* < 0.005 uncorrected with 10 continuous voxels. We found that compared to the Baduanjin group, there was a significant increase in fALFF in the periaqueductal gray, bilateral DLPFC, and temporoparietal junction in the TCC group. Compared with the TCC group, the Baduanjin group was associated with a significant fALFF increase in the left mPFC and left precuneus.

To explore the association between the fALFF changes observed above and behavioral outcomes, we also extracted the average fALFF values of the significant clusters (DLPFC and mPFC) and performed a multiple regression analysis including age, gender, and education as covariates. Results showed a significant association between the fALFF changes at mPFC and corresponding MQ (*r* = 0.48, *p* = 0.005 significant after Bonferroni correction (0.025 (0.05/2); Figure 1C), as well as a marginal association between the fALFF changes at DLPFC and corresponding MQ changes (*p* = 0.048, not significant after Bonferroni correction across the TCC and Baduanjin groups).

#### fALFF in Slow-5 Band

Pre- and post-treatment comparison of fALFF in the slow-5 band among the three groups is shown in Table 2 and Figure 1. After 12 weeks, participants in the TCC group showed significant increases in the right DLPFC compared with participants in the control group (Figure 1D). Participants in the Baduanjin group showed significant increases in the bilateral mPFC compared with participants in the control group (Figure 1E). No significant difference was found between the TCC and Baduanjin groups at the threshold we set.

To further explore the difference between Tai Chi vs. Baduanjin comparisons, we applied a relatively less conservative threshold of voxel-wise *p* < 0.005 uncorrected with 10 continuous voxels. We found that compared to the Baduanjin group, there was a significant fALFF increase in the right lateral prefrontal cortex and periaqueductal gray/pon, and a significant fALFF decrease in the left DLPFC in the TCC group.

To explore the association between the fALFF changes observed above and behavioral outcomes, we also extracted the average fALFF values of the significant clusters and performed multiple regression analysis respectively including age, gender and education as covariates across the participants in TCC and Baduanjin group. Results showed a significant association between the mPFC fALFF changes and corresponding MQ changes (*r* = 0.40, *p* = 0.02, significant after after Bonferroni correction (0.025 (0.05/2; Figure 1F). There was no significant association between DLPFC fALFF changes and corresponding MQ changes (*p* = 0.078).

####  fALFF in Slow-4 Band

No significant differences among the three groups (two exercise groups and one control group) were observed. When we applied a relatively less conservative threshold of voxel-wise *p* < 0.001 uncorrected with 10 continuous voxels, we also did not find any significant results between the three groups.

## Discussion

In this study, we investigated the effects of 12 weeks of TCC and Baduanjin exercise on fALFF changes and clinical outcome measures in older adults. We found that: (1) MQ significantly increased in both TCC and Baduanjin groups compared with the control group; (2) TCC increased fALFF in the right DLPFC in the slow-5 band and the 0.01–0.08 Hz band; and (3) Baduanjin increased fALFF in the bilateral mPFC in the slow-5 band and the 0.01–0.08 Hz band following exercise. fALFF changes at the mPFC in the slow-5 and 0.01–0.08 Hz bands showed a significant positive association with corresponding MQ changes.

Both TCC and Baduanjin are mind-body exercises consisting of meditation, breathing, and gentle movements. From the viewpoint of physical exercise, both TCC and Baduanjin are safe aerobic activities (Li R. et al., 2014; Wayne et al., 2014). Aerobic exercise has been shown to improve memory function (Flöel et al., 2010; Erickson et al., 2011; Li L. et al., 2014; Seo et al., 2014). In addition to the physical component, TCC and Baduanjin also include sustained attention, focus, and multi-tasking. Thus, the mind-body exercise component may also have positive effects on cognitive function. Our finding of a significant improvement in general memory function after 3 months of TCC and Baduanjin practice is consistent with previous studies (Chang et al., 2010; Miller and Taylor-Piliae, 2014; Zheng et al., 2015) showing positive cognitive benefits of TCC in older adults. Our study demonstrates the power of TCC and Baduanjin practice in helping older adults improve memory.

We found that compared to controls, participants in the TCC group had increased fALFF in the right DLPFC, while the participants in the Baduanjin group had increased fALFF in the bilateral mPFC in the slow-5 band and the 0.01–0.08 Hz band. Although TCC and Baduanjin are associated with different patterns compared to controls, we did not find significant differences between the TCC and Baduanjin groups at this threshold we set. However, at a less conservative threshold of voxel-wise *p* < 0.005 uncorrected with 10 continuous voxels, there was a significant fALFF increase in the bilateral DLPFC, and decrease in the left mPFC and left precuneus in the TCC group compared to Baduanjin group in the 0.01–0.08 band. We also found that compared to the Baduanjin group, there was a significant fALFF increase in right lateral prefrontal cortex compared to the Baduanjin group in the slow-5 band. These significant difference activity pattern between TCC and Baduanjin is consistent with the findings when comparing: (1) Tai Chi vs. Control; and (2) Baduanjin vs. Control. Furthermore, we also found that TCC and Baduanjin modulate the subtests of WMS-CR differently, Baduanjin can significantly increase the touch subscore compared to TCC, which further suggests that different mechanism may underlying Tai Chi Quan and Baduanjin. We speculate this difference may due to different exercise characteristics associated with the two mind-body interactions. TCC involves more complicated body movements and requires moving the trunk and all four limbs, whereas the movement involved in Baduanjin is much simpler.

In this study, we found that TCC exercise increased fALFF in the right DLPFC in the slow-5 band and the 0.01–0.08 Hz band compared to the control group. Previous studies have demonstrated that the DLPFC, a task positive region, is a key area in the cognitive control network (CCN; Miller and Cohen, 2001; Cieslik et al., 2013). The CCN is important in top-down modulation of attention–memory interactions (Corbetta and Shulman, 2002; Cole and Schneider, 2007; Chiu and Yantis, 2009; Spreng et al., 2010; Kong et al., 2013; Hwang et al., 2015; Rosen et al., 2016). Recent studies have shown that non-invasive brain stimulation techniques such as repetitive transcranial magnetic stimulation and transcranial direct current stimulation of the DLPFC enhanced memory-guided responses in a visuospatial working memory task (Balconi and Ferrari, 2012; Brunoni and Vanderhasselt, 2014; Giglia et al., 2014). These findings further confirm the DLPFC’s role in memory function.

Previous studies (Baron Short et al., 2010) found significantly increased DLPFC activation during meditation in comparison to a control task. In a recent study, investigators found that compared with control participants, TCC experts show greater functional homogeneity in the right post-central gyrus and lower functional homogeneity in the right DLPFC and the left anterior cingulate cortex. The gain in functional integration was significantly correlated with cognitive performance in TCC experts (Wei et al., 2014). In another study, investigators found that multimodal intervention including TCC exercise enhanced the ALFF in the right middle frontal gyrus/DLPFC in older adults (Yin et al., 2014). In a more recent study, we found that TCC practice significantly modulates the rsFC between the CCN and the superior frontal gyrus and ACC, and that Baduanjin modulates the rsFC between the CCN and the putamen and insula (Tao et al., 2017a). Our results are partially consistent with these findings.

We also found fALFF increases in the slow-5 band and the 0.01–0.08 Hz band in the Baduanjin group at the mPFC. This change was significantly associated with memory function changes. The mPFC is associated with the highest baseline metabolic activity at rest (Gusnard et al., 2001) and is a key region in the default mode network (DMN; Li L. et al., 2014). The mPFC identified in the present study overlaps with the findings observed in previous studies on the impact of physical activity and meditation on cognitive functions (Flöel et al., 2010; Hasenkamp and Barsalou, 2012; Tang and Posner, 2014; Tamura et al., 2015).

The mPFC is known to undergo both structural and functional changes with aging (Gutchess et al., 2007; Hurtz et al., 2014; van de Vijver et al., 2014). Research suggests that the mPFC’s function is related to different aspects of social cognitive processing (Amodio and Frith, 2006), which involves action monitoring (Barch et al., 2001), self-knowledge (Macrae et al., 2004), person perception, mentalization (Grèzes et al., 2004), and outcome monitoring (Camille et al., 2004). Studies also found the mPFC is involved in the encoding and retrieval of memory (van Kesteren et al., 2010, 2012; Brod et al., 2013). In a previous study based on the same data, we found that TCC and Baduanjin (at a less conservative threshold) can increase the rsFC between the hippocampus and mPFC (Tao et al., 2016). Taken together, our result suggests that Baduanjin may improve memory function through the mPFC and associated brain networks, such as the hippocampus.

In this study, we found that the slow-5 frequency band and the low-frequency band (0.01–0.08 Hz) showed fALFF changes after TCC and Baiduanjin practice, while no significant differences were observed in the slow-4 band. This suggests that the memory-relevant changes induced by 3 months of TCC and Baduanjin practice are specifically reflected difference low-frequency band. In a previous study, Han et al. (2011) investigated changes of ALFF and fALFF in patients with MCI between the slow-4 and slow-5 bands. They found significant differences in fALFF between MCI patients and controls only in the slow-5 band. The pattern of intrinsic functional connectivity is sensitive to specific frequency bands. Chao-Gan and Yu-Feng (2010) found that the low-frequency range (0.01–0.08 Hz) may better reveal the DMN. Zuo et al. (2010) also shown that Slow-5 (0.01–0.027 Hz) amplitudes at low frequency were more dominant within ventromedial prefrontal cortices. Consistent with these results, our findings also predominantly locate at the frontal area in Slow-5 band and low-frequency range. Further studies are needed to confirm and expand our findings.

The current study has several limitations. First, the sample size is relatively small. Second, both TCC and Baduanjin are considered mind-body exercises. Therefore, our design could not disentangle the effect of physical activity vs. mental exercise, and was unable to identify the crucial components of the exercise affecting memory improvement and brain functional fluctuation changes. Further research is needed to compare the effects of exercise, meditation, TCC and Baduanjin directly. Third, we did not record the participants’ original physical activity habits, and only gave them an introduction to keep their original physical activity habits throughout the duration of the experiment. Further study should record the participants original physical activity habits for more accurate documentation of their activity intensity.

## Conclusion

In this study, we found that 12 weeks of intensive group TCC/Baduanjin practice can significantly modulate fALFF in different frequency bands and improve memory function. TCC increased fALFF in the slow-5 band and the 0.01–0.08 Hz band in the DLPFC, and Baduanjin increased fALFF in the slow-5 band and the 0.01–0.08 Hz band in the mPFC. fALFF changes in the mPFC in the slow-5 band and 0.01–0.08 Hz band were also correlated with general memory improvement. Our results imply that TCC and Baduanjin may work through different brain mechanisms due to differences in the characteristics and complexity of their respective regimens, but both exercises may hold the potential to prevent age-related memory decline.

## Ethics Statement

The Medical Ethics Committee in the Affiliated Rehabilitation Hospital of Fujian University of Traditional Chinese Medicine approved all study procedures. The experiment was performed in accordance with approved guidelines. All participants signed a written consent. This study was registered on the Chinese Clinical Trial Registry (ChiCTR-IPR-15006131).

## Author Contributions

LC: experimental design; JK: analysis and manuscript preparation; JT: experimental design, data analysis and manuscript preparation; GZ: data analysis; JL and XX: data collection and data analysis; WL, JW, ML, ZW and KH: data collection; XC and NE: manuscript preparation. All authors contributed to manuscript draft and have read and approved the final manuscript.

## Conflict of Interest Statement

JK has a disclosure to report; holding equity in the startup company MNT, but declares no conflict of interest. The other authors declare that the research was conducted in the absence of any commercial or financial relationships that could be construed as a potential conflict of interest.

## Footnotes

^1^http://www.chictr.org.cn/index.aspx

^2^http://www.fil.ion.ucl.ac.uk/spm

^3^http://www.restfmri.net

## Abbreviations

AD: Alzheimer’s disease
ALFF: amplitude of low frequency fluctuations
aMCI: amnestic mild cognitive impairment
BDI: Beck depression inventory
CCN: cognitive control network
CSF: cerebrospinal fluid
DLPFC: dorsolateral prefrontal cortex
DPARSF: Data Processing Assistant for Resting-State fMRI
fALFF: fractional amplitude of low-frequency fluctuations
FWHM: full-width at half maximum
LFF: low frequency fluctuations
MCI: mild cognitive impairment
MDD: major depressive disorder
MMSE: Mini-Mental State Exam
mPFC: medial prefrontal cortex
MPRAGE: magnetization-prepared rapid gradient echo
PD: Parkinson’s Disease
SAD: social anxiety disorder
TCC: Tai Chi Chuan
WMS-CR: Wechsler Memory Scale–Chinese Revision.
